# Dominin and Segon Form Multiprotein Particles in the Plasma of Eastern Oysters (*Crassostrea virginica*) and Are Likely Involved in Shell Formation

**DOI:** 10.3389/fphys.2019.00566

**Published:** 2019-05-15

**Authors:** Qinggang Xue, Jean-Philipe Beguel, Jerome La Peyre

**Affiliations:** ^1^Key Laboratory of Aquatic Germplasm Resource of Zhejiang, Zhejiang Wanli University, Ningbo, China; ^2^Laboratory for Marine Fisheries Science and Food Production Processes, Qingdao National Laboratory for Marine Science and Technology, Qingdao, China; ^3^School of Animal Sciences, Louisiana State University Agricultural Center, Baton Rouge, LA, United States

**Keywords:** bivalve, mollusk, hemolymph plasma, shell repair, 2-DE, metal binding protein

## Abstract

Dominin and segon are two proteins purified and characterized from the plasma of eastern oysters *Crassostrea virginica*, making up about 70% of the total plasma proteins. Their proposed functions are in host defense based on their pathogen binding properties and in metal metabolism based on their metal binding abilities. In the present study, the two proteins were further studied for their native states in circulation and extrapallial fluid and their possible involvement in shell formation. Two-dimensional electrophoresis confirmed that the oyster plasma was dominated by a few major proteins and size exclusion chromatography indicated that these proteins were present in circulation in a morphologically homogenous form. Density gradient ultracentrifugation in Cesium Chloride isolated morphologically homogenous particles of about 25 nm in diameter from the plasma and extrapallial fluids. Polyacrylamide gel electrophoresis identified dominin, segon and an unidentified protein as the principal components of the particles and the three proteins likely formed a multiprotein complex that associated to form the particle. Additionally, three major proteins extracted from shell organic matrix were identified based on the apparent molecular weight in SDS-PAGE to correspond to the three major proteins of plasma and protein particles. Moreover, the hemocyte expression of dominin and segon genes measured by real-time RT-PCR increased significantly upon the initiation of shell repair and were significantly greater in younger oysters. These findings suggest that dominin and segon form protein particles by association with each other and perhaps some other major plasma proteins and play a significant role in oyster shell formation.

## Introduction

Hemolymph plays a pivotal role in gas exchange, nutrient distribution, elimination of wastes and internal defense in bivalve mollusks (Cheng, 1996; Gosling, 2003). These hemolymph functions are performed by both a cellular component, the hemocytes, and a non-cellular component, the colorless plasma. Studies on hemocytes have been extensively carried out during the last several decades mainly because of their important role in host defense (La Peyre et al., 1995; Xue and Renault, 2001; Canesi et al., 2002; Jemaà et al., 2014; Allam and Raftos, 2015; Gervais et al., 2018). Studies have also revealed that the plasma of bivalves may contain 100s of proteins (Kania et al., 1980; Suhr-Jessen and Rasmussen, 1988; Simonian et al., 2009a,b; Oliveri et al., 2014; Fernández-Boo et al., 2016; Franco-Martínez et al., 2018). The identification and functional characterization of bivalve plasma protein is, however, largely limited to proteins of lower abundance involved in bivalve immunity such as antimicrobial peptides and proteins (Charlet et al., 1996; Mitta et al., 1999; Xue et al., 2004, 2010), lectins and other recognition proteins (Tunkijjanukij et al., 1998; Minamikawa et al., 2004; Takahashi et al., 2008; Adhya and Singha, 2016), and protease inhibitors (Xue et al., 2006, 2009; La Peyre et al., 2010). On the other hand, proteins most abundant or “major” in bivalve plasma lack attention.

Several types of major proteins have been identified in the plasma and tissue fluids of bivalves. In heterodont bivalves such as the soft-shell clam *Mya arenaria*, a phosphoprotein extremely rich in aspartic acid, phosphoserine and histidine residues is present in circulating and intercellular plasma as a particle of 30–40 nm in diameter (Marsh and Sass, 1984, 1985; Marsh, 1986b, 1990). The phosphoprotein and perhaps the protein particles are formed in hemocytes. They have calcium binding capacity and are believed to play a role in shell mineralization. In the blue mussel *Mytilus edulis*, belonging to another bivalve subclass the Pteriomorphia, a histidine rich glycoprotein (HRG) of 63 kDa makes up about 60% of the total plasma protein (Nair and Robinson, 1999). HRG, also called heavy metal binding protein HIP in sequence databases or serum protein band 1 (SBP1, Renwrantz et al., 1998; Schneeweiss et al., 2002), is secreted by mussel hemocytes and is found in plasma as diverse polymers (Renwrantz and Werner, 2008). Its function is related to the transfer of heavy metals to the kidney for detoxification (Renwrantz et al., 1998; Nair and Robinson, 1999, 2001a,b; Devoid et al., 2007; Robinson, 2013). This protein is also found in blue mussel extrapallial fluid where it is believed to be involved in shell formation and named EP (Hattan et al., 2001; Yin et al., 2005). Interestingly, HRG was described as the most abundant plasma protein in six bivalve species including the eastern oyster *Crassostrea virginica* (Abebe et al., 2007). However, this conclusion is inaccurate as oyster plasma contains a different type of major protein.

The major plasma protein of the Pacific oyster (*Crassostrea gigas*) is cavortin which was originally described by Scotti et al. (2001, 2007) and later named extracellular superoxide dismutase (i.e., Cg-EcSOD) by Gonzalez et al. (2005) because of its homology to Cu/Zn superoxide dismutase. Cavortin, however, lacks superoxide dismutase activity (Scotti et al., 2007). It has thus mainly been studied for its putative role in host defense based on its binding to pathogen-associated molecular patterns (PAMPs) and its expression in response to viral, bacterial, and protozoan challenges (Gonzalez et al., 2005; Duperthuy et al., 2011; Green et al., 2014; Normand et al., 2014; Liu et al., 2016). Proteins with sequences deduced from cDNA and similar to that of cavortin or part of cavortin were also identified in the Sydney rock oyster, *Saccostrea glomerata* (Sg-EcSOD, Green et al., 2009) and the European flat oyster, *Ostrea edulis* (Oe-EcSOD, Morga et al., 2011). The plasma of the New Zealand green-lipped mussel, *Perna canaliculus*, also has a major protein, pernin, which contains three CuZn-SOD domains (Scotti et al., 2001) but lacks SOD activity and can be purified by ultracentrifugation as protein particles and bind multiple metal ions such as Cavortin (Scotti et al., 2001, 2007).

Mollusk shell formation involves biomineralization, which is initiated by the generation of an organic matrix that makes up the principal composition of the outmost layer of the shell (i.e., the periostracum). This is followed by the nucleation and growth of calcium carbonate crystals to form the two other layers of the shell, the prismatic layer in the middle and the innermost foliated layer (Addadi et al., 2006; Gong et al., 2008; Marin et al., 2012; Suzuki and Nagasawa, 2013). As mentioned earlier, the calcium-binding phosphoprotein particles of heterodont bivalve plasma are believed to serve as the source and transport vehicle for the calcium ions during shell mineralization (Marsh, 1986a). In blue mussel, the drastic change of secondary structure of the purified major extrapallial fluid protein EP, which is also the major plasma protein, upon addition of calcium ions to a threshold concentration supported EP involvement in shell matrix formation (Hattan et al., 2001; Yin et al., 2005). In the eastern oyster, two shell matrix proteins were found in plasma and hemocytes by western blotting (Johnstone et al., 2008). It is therefore clear that some hemocyte-secreted plasma major proteins are associated with calcium ion transport and shell formation.

Two major proteins, dominin and segon, have recently been purified and characterized from eastern oyster plasma by preparative SDS-PAGE (Itoh et al., 2011; Xue et al., 2012). Dominin is a 32.5 kDa protein under reduced SDS-PAGE and is present in plasma in multiple molecular forms. It undergoes post-translational modification including glycosylation and likely phosphorylation from its 19,389 Da molecular weight calculated from its amino acid sequence. The protein has a Cu/Zn superoxide dismutase domain but no enzyme activity and its sequence is similar to cavortin (Cg-EcSOD) of Pacific oysters. Segon, on the other hand, is a novel protein of 39 kDa under reduced SDS-PAGE with a calculated molecular weight of 30,484 Da from its amino acid sequence that shows no significant similarity with any known proteins. According to SDS-PAGE analysis, dominin (cavortin in Pacific oysters) and segon account for about 50 and 20% of the total plasma proteins respectively of eastern oysters and other oyster species including Pacific oysters, Suminoe oysters (*Crassostrea ariakensis*) and European flat oysters (Xue et al., 2012). Considering both proteins are secreted by hemocytes, are abundant in extrapallial fluid and bind multiple metal ions including calcium, their potential role in shell formation was investigated. In addition, the native state of these major proteins in eastern oyster plasma was characterized. Results of the research could generate important information about the functions of these abundant proteins in oysters and perhaps of related proteins in other bivalve species.

## Materials and Methods

### Oyster Plasma and Extrapallial Fluid Samples

Eastern oysters, 10–15 cm in shell height, were obtained from Louisiana Sea Grant Oyster Research and Demonstration Farm off Grand Isle, Louisiana, in the northern Gulf of Mexico in January 2012. The oysters were maintained in a recirculating seawater system at salinity of 15 ppt at 15°C for 1 week prior to hemolymph collection. Oyster hemolymph was withdrawn from the adductor muscle sinus using a 3 ml syringe equipped with a 25-gauge needle through a notch on the dorsal side of the shell made with a Skil 4-1/2″ angle grinder. Hemolymph from individual oysters was pooled and centrifuged for 15 min at 300 *g*, 4°C. After centrifugation the supernatant was collected as plasma and stored at -20°C before analyses. Oyster extrapallial fluids were collected from the shell cavity through a notch on the posterior side of each oyster. Extrapallial fluids from individual oysters were pooled, centrifuged as the hemolymph samples and the supernatants were stored at -20°C before analyses. Protein concentrations of plasma and extrapallial fluid samples were measured using the Micro BCA Protein Assay Reagent Kit from Pierce Biotech (Rockford, IL, United States) with bovine serum albumin as a standard.

### Two-Dimensional Gel Electrophoresis

Oyster plasma was profiled by two-dimensional gel electrophoresis (2-DE) using immobilized pH gradient gel strips for isolectric focusing (IEF) for the first dimensional separation and then SDS-PAGE for the second dimensional separation. Oyster plasma samples, 300 μg per sample, were processed using the Bio-Rad ReadyPrep^TM^ 2-D Cleanup Kit according to the manufacturer’s instruction (Bio-Rad, Hercules, CA, United States) and then dissolved in 200 μl of 2-DE sample buffer containing 7 M urea, 2 M thiourea, 1% amberlite MB-150, 4% CHAPS, 0.5% Ampholyte (3–10, Bio-Rad), 65 mM DTT, and 10% isopropanol. The dissolved sample solution was used to rehydrate a pH 3–10 ReadyStrip^TM^ IPG strip of 11 cm (Bio-Rad) for 12 h in a rehydration/equilibration tray placed in a Bio-Rad Protean^®^ IEF Cell under the passive rehydration program. The IPG strip was then transferred into a focusing tray and IEF was performed in the Bio-Rad Protean^®^ IEF Cell with the preset program. After IEF, the IPG strip was equilibrated in two steps, 15 min each, first in equilibration buffer 1 (6 M urea, 2% SDS, 0.375 M Tris-HCl pH 8.8, 20% glycerol and 130 mM DDT) and then in equilibration buffer 2 (6 M urea, 2% SDS, 0.375 M Tris-HCl pH 8.8, 20% glycerol, and 135 mM iodoacetamide), after which it was placed on top of a Bio-Rad 8–16% gradient Criterion^TM^ precast SDS-PAGE gel for the second dimension separation. The IPG strip mounted gel was run under constant 200 V voltage for 50 min in a Criterion^TM^ cell with Tris-HCl buffer. Proteins in the gel were stained using the Bio-Safe^TM^ Coomassie Staining solution from Bio-Rad according to the manufacturer’s instruction.

### Size Exclusion Chromatography

Oyster plasma was fractionated using size exclusion chromatography in a 1.6 cm × 70 cm Superdex 200 column (Amersham Biosciences, Piscataway, NJ, United States). Oyster plasma was concentrated by ultrafiltration using a Ultracentrifugal Unit (MWCO 10 kDa, Millipore, Billerica, MA, United States) and centrifugation at 2,500 *g*, 4°C, and 1 ml of concentrated oyster plasma containing 30 mg proteins was applied to the column connected to a BioLogic DuoFlow Pathfinder 20 System from Bio-Rad. The column was eluted with 0.5 M NaCl in 0.02 M Tris-HCl, pH 8.0 at an elution rate of 0.5 mL/min. The elution was monitored for absorbance at 240 nm with a QuadTec UV/Vis detector and collected in 3 ml fractions.

### Oyster Plasma Ultracentrifugation

Oyster plasma samples were centrifuged separately at 100,000, 200,000 or 300,000 *g*, 4°C, for 30, 60, 90, or 120 min using a Beckman L8-70M Ultracentrifuge with a Type 70 Ti Fixed Angle Rotor (Beckman Coulter Life Sciences, Indianapolis, IN, United States). After centrifugation, the total protein amount in the supernatants was determined by multiplying the supernatant protein concentration with the total supernatant volume. The percent precipitated protein was then calculated according to the following formula: % precipitated proteins = [(original total protein-supernatant total protein)/original total protein] × 100%. The centrifugation and protein measurements were carried out in triplicate for each time period and centrifugal force.

### Protein Particle Purification and Electron Microscopy

Plasma and extrapallial fluid samples were centrifuged at 300,000 *g*, 4°C, for 120 min using a Beckman L8-70M Ultracentrifuge with a Type 70 Ti Fixed Angel Rotor. The protein pellets were dissolved in 1.39 g/ml CsCl solution in 0.02 M Tris-HCl, pH 8.0. The solution was centrifuged at 5,000 *g*, 4°C for 15 min to remove insoluble particles and the supernatant was then centrifuged at 200,000 *g*, 4°C for 20 h using a Beckman L8-70M Ultracentrifuge with a SW 55 Ti Swinging-Bucket rotor. After centrifugation, purified protein particles were collected from the turbid layer in the centrifuge tube using a 1 ml syringe equipped with a 25-gauge needle. The collected samples were stored at -20°C before analyses for morphology by transmission electron microscopy and for protein composition by polyacrylamide gel electrophoresis. The purified protein particles were observed after negative stain with 0.5% uranyl acetate at the Louisiana State University Shared Instrumentation Facility in Baton Rouge, Louisiana using a JEOL-1400 Transmission Electron Microscope (JEOL USA, Inc., Peabody, MA, United States).

### Polyacrylamide Gel Electrophoresis

Oyster plasma proteins and purified protein particles were analyzed to determine their protein composition using native polyacrylamide gel electrophoresis (native-PAGE) and SDS-PAGE. In the native-PAGE, a 4–15% linear gradient polyacrylamide gel of 16 cm in separation gel height was hand-cast with the discontinuous Laemmli buffer system without SDS (Laemmli, 1970). The protein samples were prepared in the native PAGE sample buffer purchased from Bio-Rad and the electrophoresis was run in a Protean^®^ II xi Cell under constant 30 mA current and cooling using circulating ice water. Proteins in the gel were stained using the Bio-Safe^TM^ Coomassie Stain from Bio-Rad according to the manufacturer’s instruction. Two Coomassie blue stained protein bands were cut from the native PAGE gel and the proteins contained in the gel pieces were eluted separately using a Bio-Rad Model 422 Electro-Eluter and concentrated by ultrafiltration using MWCO 10 kDa Ultracentrifugal Unit) as described above. After concentration, the eluted protein samples along with the purified protein particles were analyzed by SDS-PAGE in a 10% acrylamide/bis gel using a Bio-Rad Protean mini Cell and stain with Bio-Safe^TM^ Coomassie Stain.

### Shell Matrix Protein Extraction and Analysis

Soluble matrix proteins were extracted from the newly formed shells of 12-month-old oysters obtained from Louisiana Sea Grant Oyster Research and Demonstration Farm. Briefly, partially calcified oyster shell pieces were collected from the edges of both valves using forceps. Oyster shell pieces were rinsed three times with water and then cleaned by immersion in 0.5 M NaOH overnight at 1:100 (w/v) with periodical hand shaking. After three rinses in deionized water, the cleaned shell pieces were transferred into 1.5 M acetic acid at a ratio of 1 g of shell pieces in 20 ml of acetic acid solution and slowly stirred for 72 h at 4°C using a magnetic stirrer. The supernatants were collected and concentrated by ultrafiltration using a MWCO 10 kDa Ultracentrifugal Unit. After concentration, the acetic acid extracted matrix proteins were rinsed in deionized water and then analyzed using SDS-PAGE in 10% polyacrylamide gel using a Bio-Rad Protean mini cell and Coomassie blue staining.

### Oyster Shell Damage Study Design

**FIGURE 1:**
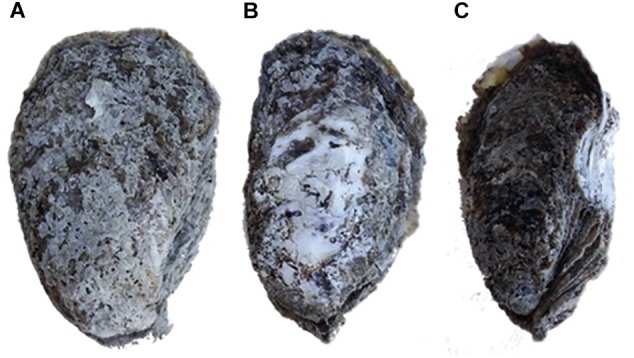
Illustration of oyster shell damage treatments. **(A)** Control oyster; **(B)** abraded oyster; **(C)** notched oyster.

Sixty-five 30-month- old eastern oysters, 12.4 ± 1.1 cm in shell height, were obtained from Louisiana Sea Grant Oyster Research and Demonstration Farm in Grand Isle in December 2013. The oysters were transferred to a recirculating seawater system at salinity of 20 and at 17 ± 1°C, under the same conditions as when collected, and were fed daily with 0.2 ml of Shellfish Diet 1800^®^ (Reed Mariculture) per oyster. After 4 days of acclimation, the hemolymph of five oysters were sampled as described earlier and individually placed into tubes in an ice-bath. Hemocyte densities were determined using a hemocytometer and hemolymph volumes containing 10^7^ hemocytes were transferred into new tubes. The new tubes were centrifuged at 400 *g* at 4°C for 10 min and hemocyte pellets were stored at -80°C until processed for RNA extraction. The 60 oysters left over were then divided haphazardly into three groups of 20 oysters designated as notched, abraded or control. The notched oysters had the edges of both valves in the middle of the dorsal side ground for 20 s to form a notch about 1.5 cm long that opened into the shell cavity (Figure 1). The shell surfaces of the right valves of the abraded oysters were ground for 20 s to determine the potential effect of grinding vibration. The control oysters in the third group were treated the same way as the oysters in the other two groups except their shells were not ground. The hemocyte pellets of five oysters per group were collected on days 1, 2, 4, and 8 and stored at -80°C until processed for RNA extraction.

### Reverse Transcription Quantitative Real Time PCR (RT-qPCR)

Dominin and segon mRNA levels in oyster hemocytes were measured by RT-qPCR. Total RNAs were extracted from hemocyte samples using RNeasy^®^ Mini Kit and treated with RNase-Free DNase Set (QIAGEN) according to manufacturer’s instructions to prevent DNA contamination. RNA concentrations were estimated from the A260 measured using a Nanodrop spectrometer (Thermo Scientific) with 1 OD = 40 μg/ml RNA. The RNA quality was analyzed using RNA nanochips and Agilent RNA 6000 Nano Reagents (Agilent Technologies) according to manufacturer’s instructions. The Integrity Numbers (RIN) of the RNAs used in the study were greater than 9.40. Total cDNAs were synthesized with 750 ng of total RNAs in a final volume of 20 μl using Omniscript RT Kit with Oligo-dT Primers and RNase Inhibitor (QIAGEN) according to manufacturer’s instructions.

RT-qPCR assays were performed in a Bio-Rad CFX96^TM^ Real-Time C1000 Thermal Cycler using the synthesized total cDNA as template and primers specific for dominin and segon designed using Primer3 (Rozen and Skaletsky, 2000). The gene specific primers used in the assays were the same as those reported previously (Itoh et al., 2011; Xue et al., 2012). The reactions were carried out in a total volume of 10 μl containing 4 μl of 1:30 diluted cDNA, 0.5 μl of each primer at 10 μM and 5 μl of SsoFast^TM^ EvaGreen^®^ Supermix (Bio-Rad). The cycling conditions consisted of Taq polymerase activation for 30 s at 95°C, followed by 40 cycles of denaturation at 95°C for 15 s and annealing/elongation at 60°C for 45 -s. For each sample, a melting curve program was applied from 60 to 95°C by increasing temperature at a ramp rate of 1%. PCR amplification efficiency (E) of each primer pair was determined from a serial dilution (1:10–1:10000) of a reference cDNA sample made by pooling equal amount of digestive gland cDNA from 20 abraded oysters according to the following formula E = 10(-1/slope). Oyster elongation factor I (EFI) and 18S rRNA genes were used as internal references and their steady-state expression was confirmed by PCR amplifications that showed a coefficient of variance (CV) of less than 5% (Araya et al., 2008; Siah et al., 2008). Negative reverse-transcription controls were run by replacing cDNA template with total RNA samples after DNase treatment to ensure the absence of DNA carryover. Dominin and segon mRNA levels were determined relative to the geometric mean of the two reference genes using the ΔΔCt method (Pfaffl, 2001). Measurements were done in triplicate. Data were analyzed using a two-factor (treatment, time) analysis of variance (ANOVA) followed by Tukey’s test. The correlation of expression between dominin and segon was analyzed using Spearman’s Rank Order Correlation method.

### Comparison of Dominin and Segon Gene Expression in Younger and Older Oysters

Five 6-month-old eastern oysters were obtained from Louisiana Sea Grant Oyster Research and Demonstration Farm in Grand Isle in December 2013 and transferred to the same recirculating seawater system as the 30-month-old oysters used in the shell repair study. After 4 days of acclimation, 10^7^ hemocytes were collected from each oyster. Dominin and Segon gene expression in hemocytes of the 4-day acclimated 6- and 30-month-old oysters were then compared as described earlier. Data were analyzed by a rank sum test.

## Results

### Oyster Plasma Protein Profile in 2-DE

**FIGURE 2:**
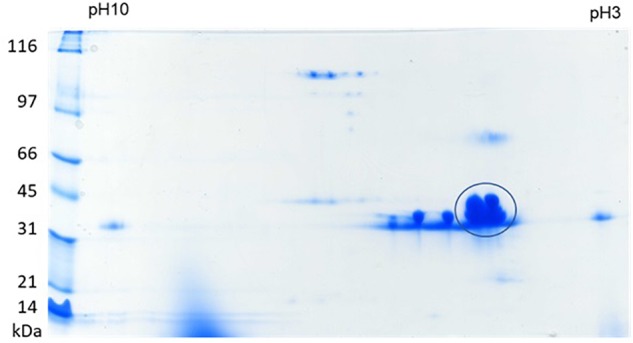
2-DE of eastern oyster plasma proteins. Analysis was done by isoelectric focusing using a 11 cm IPG strip of pH 3–10 for the first dimension and SDS-PAGE on an 8–16% gradient polyacrylamide gel as the second dimension. Proteins were visualized by staining the gel in Coomassie blue.

To explore whether the dominant protein bands present in previously reported SDS-PAGE analysis represented the overlap of multiple proteins, 2-DE of the plasma of eastern oysters was carried out. About 40 protein spots were identified in the eastern oyster plasma using 2-DE separation and Coomassie blue staining (Figure 2). Except for a few proteins that were extremely acidic or alkaline and several proteins that were found to have a molecular weight of about 100 kDa, most oyster plasma proteins were visualized in the acidic to neutral pH range and at the molecular weight location around 40 kDa. The most intense spot appeared to cover 4–5 protein molecules with the pI around 5 and the molecular weight around 43 kDa, which were not clearly separated by either IEF or SDS-PAGE. Two less intense protein spots showed similar molecular size and abundance but differed in pI.

### Chromatogram of Oyster Plasma

**FIGURE 3:**
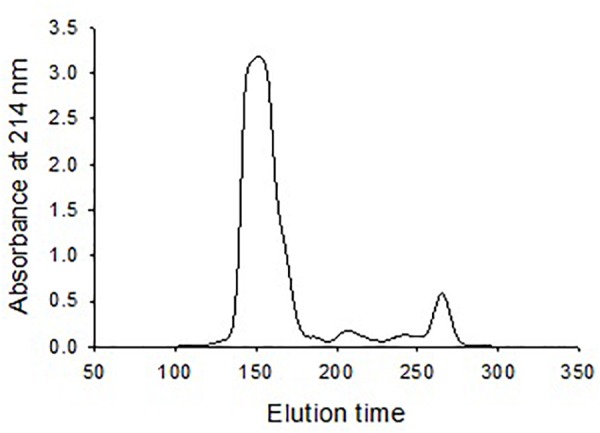
Chromatogram of oyster plasma protein fractionated by size exclusion chromatography in a 1.6 cm × 70 cm Superdex 200 column. One milliliter of plasma sample containing 30 mg total proteins was loaded and the column was eluted at an elution rate of 0.5 ml/min.

Size exclusion chromatography was used to investigate the morphological heterogenicity of the eastern oyster plasma proteins in the native status. Size exclusion chromatography, also called gel filtration, separates molecules or particles based on the size and morphology. Molecules or particles similar in size and morphology occur in a same fraction, which exhibits as single absorbance peak in the chromatogram, and the fractions are recovered in an order that the largest molecule come first and the smallest the latest. After chromatography, the eastern oyster plasma proteins were fractionated into 3–4 absorbance peaks (Figure 3). Most proteins were recovered in the first and the biggest eluted peak.

### Oyster Plasma Protein Ultracentrifugation

**FIGURE 4:**
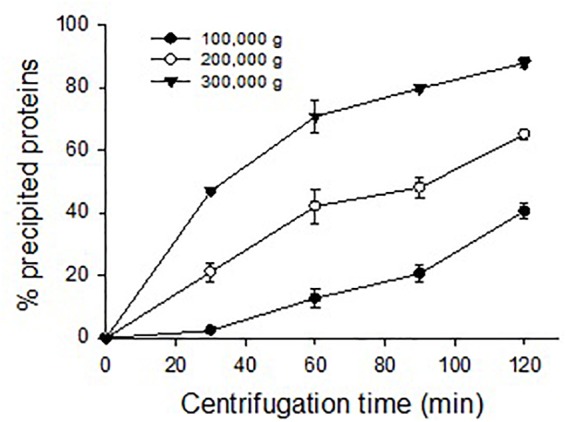
Precipitation of oyster plasma proteins by ultracentrifugation under different centrifugal force and time.

Eastern oyster plasma samples were centrifuged at 100,000, 200,000, or 300,000 *g* and for 30, 60, 90, or 120 min. The precipitated proteins formed a gelatinous transparent pellet at a rate that was proportional to the applied centrifugal force and period (Figure 4). Centrifugation at 100,000 *g* for 120 min resulted in the precipitation of about 50% of total plasma proteins. As the centrifugal force increased a similar precipitation was achieved in less time with 90 min at 200,000 *g* or 30 min at 300,000 *g*. When centrifuged at 300,000 *g* for 120 min, ∼90% of total plasma proteins were sedimented. The precipitated protein pellets were easily dissolved in Tris buffer and the solution was transparent.

### Purification and TEM Morphology of Protein Particles From Oyster Plasma and Extrapallial Fluids

**FIGURE 5:**
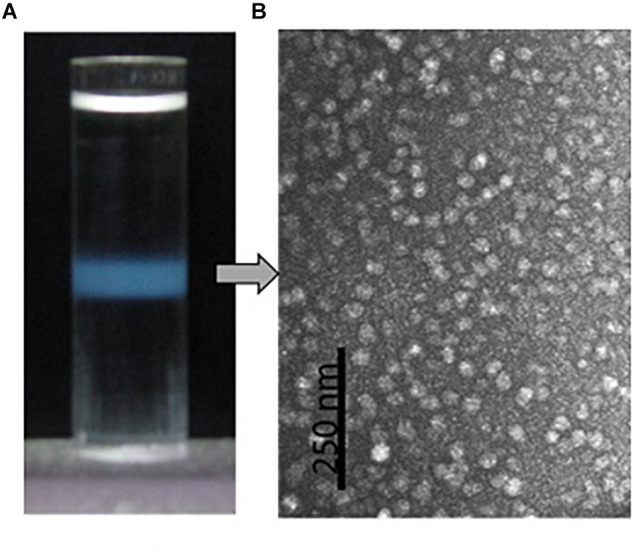
Purification by density gradient centrifugation and morphological observation by transmission electron microscopy of the oyster multiprotein particles. **(A)** Protein particles (turbid zone) in the centrifuge tube after isopycnic centrifugation at 200,000 *g*, 4°C for 20 h in 1.39 g/ml CsCl solution. **(B)** Morphology of the purified multiprotein particles under transmission electron microscope with negative stain in 0.5% uranyl acetate.

Isopycnic centrifugation in the CsCl medium was used to purify the protein particles from the oyster plasma and extrapallial fluids. Isopycnic centrifugation separates particles according to their buoyant density, which reflects the particles’ size and morphology. In the centrifugation, the CsCl solution forms a linear gradient under centrifugal force in a prolonged period. At the same time, particles originally suspended in the CsCl solution gradually reach an equilibrium at the zone where the density of the medium equals the buoyant density of the particles to be purified. A sharply edged thin turbid layer occurred in the middle of the centrifuge tube after isopycnic ultracentrifugation of the gelatinous protein pellets that was obtained by ultracentrifugation at 300,000 for 90 min and dissolved in 1.39 g/ml CsCl solution (Figure 5A). The contents of the turbid layer were examined under transmission electron microscope after negative stain with uranyl acetate. Particles of relative homogeneity in morphology and about 25 nm in diameter were observed (Figure 5B). No apparent differences in morphology were found between the protein particles obtained from the plasma and those from the extrapallial fluids.

### Protein Composition of Purified Protein Particles

**FIGURE 6:**
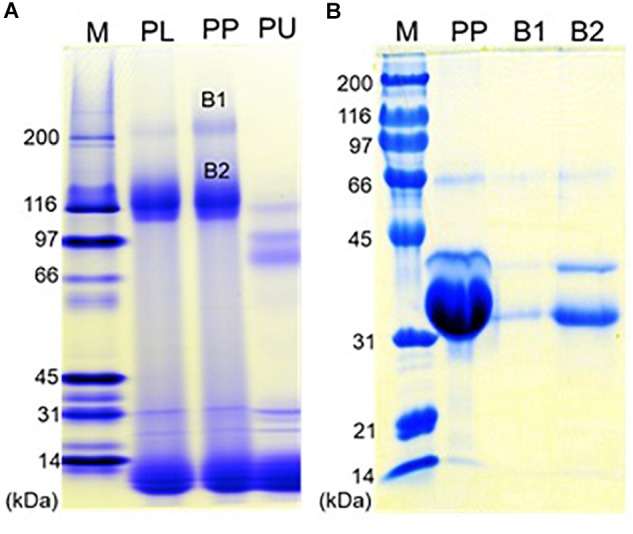
Polyacrylamide gel electrophoresis of the oyster plasma, purified multiprotein particles, and protein aggregates. **(A)** Native-PAGE of oyster plasma (PL), purified plasma protein particles (PP) and 6 M urea treated purified particles (PU) in a 16 cm tall gel of 4–15% gradient. **(B)** SDS-PAGE of purified plasma protein particles (PP) and the two protein aggregates from the native gel (B1 and B2) in a 10% gel. Lane M received Bio-Rad’s broad range protein molecular standards. Proteins were visualized by staining the gel in Coomassie blue.

Native-PAGE of eastern oyster plasma and purified protein particles in a 4–15% gradient gel visualized two intense protein bands of about 110 and 200 kDa respectively, and multiple other protein bands of <30 kDa (Figure 6A). When the protein particle samples were treated with 6 M urea at room temperature for 1 h, the 200 kDa band disappeared and the 110 kDa band became significantly less intense while two new protein bands appeared. After being cut and eluted from the polyacrylamide gel, the 200 and 110 kDa bands were analyzed separately in SDS-PAGE and both were observed to contain three similar proteins with the estimated molecular weight of about 33, 39, and 70 kDa respectively (Figure 6B). The 33 and 39 kDa proteins had the same molecular weight as dominin and segon respectively while the 70 kDa band represents another major plasma protein yet to be identified.

### Protein Composition of Newly-Grown Shells

**FIGURE 7:**
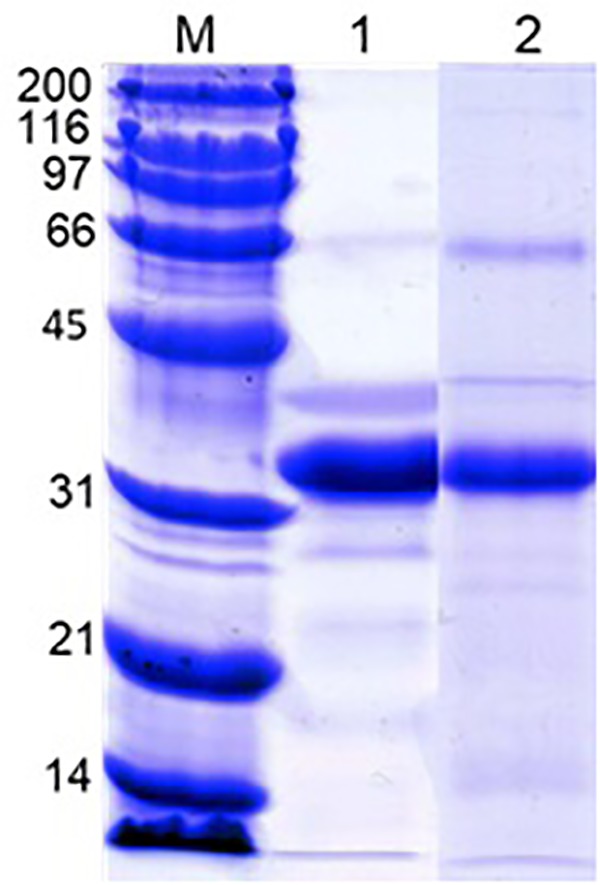
SDS-PAGE of the oyster plasma and oyster shell matrix extracts. Lane 1: oyster plasma. Lane 2: shell matrix proteins extracted from newly-grown shell using acetic acid. Analysis was done in 10% gel and proteins were visualized by staining the gel in Coomassie blue. Lane M received Bio-Rad’s broad range protein molecular standard.

Analysis of acetic acid-soluble shell matrix proteins using SDS-PAGE revealed multiple proteins. Among them three major bands showed similar migration patterns as the three major proteins in the oyster plasma and those visualized from the 110 and 200 kDa native-PAGE protein bands. However, the relative abundance of the protein apparently corresponding to the unidentified third major protein increased in the extracted shell matrix proteins. Additionally, several less intense protein bands unshared between the plasma and the shell matrix extracts were seen in both samples (Figure 7).

### Dominin and Segon Gene Expressions Following Shell Grinding

**FIGURE 8:**
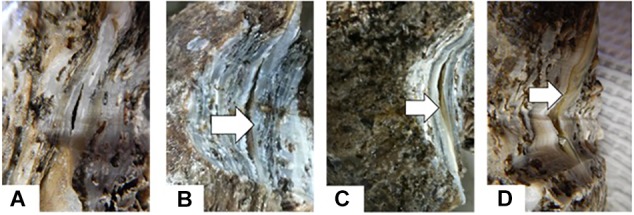
New Shell formation (arrows) two **(B)**, four **(C)** and six **(D)** days after notching eastern oysters on day 0 **(A)**.

**FIGURE 9:**
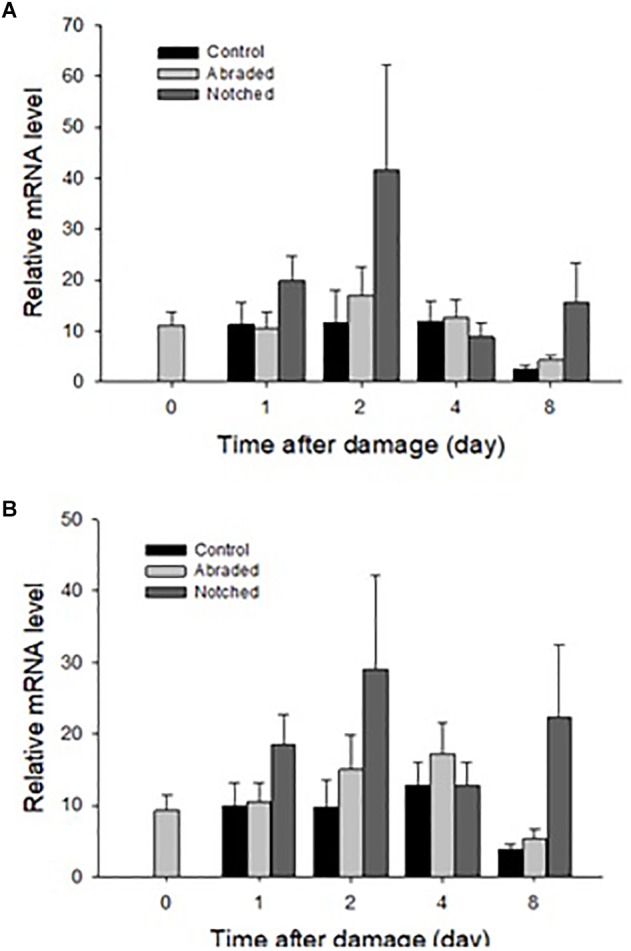
Relative mean ± standard errors expression of dominin **(A)** and segon **(B)** genes in eastern oyster hemocytes (*n* = 5). Dominin and segon mRNA levels relative to EF1 and S18 gene transcripts were measured using quantitative real-time PCR.

New shell was observed in the notched oysters 2 days after grinding their dorsal side and exposing the shell cavity. The amount of shell formed increased from day 2 to day 8 in most of the notched oysters (Figure 8). Dominin mRNA levels were elevated in hemocytes of the notched oysters with the highest level being detected on day 2 (Figure 9A); Two-factor ANOVA followed by Tukey *post hoc* test indicated significant effects of “treatment” (*P* = 0.029) and “time” (*P* = 0.009) with no interaction on transcript levels. Dominin mean mRNA level in notched oysters was significantly greater, 2.32 times, than in control oysters (*P* = 0.022) while it was 1.9 times that of abraded oysters (*P* = 0.298). In addition, dominin mean mRNA level on day 8 was significantly lower than on day 1 (*P* = 0.030) and 2 (*P* = 0.010). Segon mRNA levels were also elevated in hemocytes of the notched oysters and the highest level was detected on day 2 as with dominin (Figure 9B). Two-factor ANOVA followed by Tukey *post hoc* test only indicated a significant effect of “treatment” (*P* = 0.028) on Segon mRNA levels. Segon mean mRNA level in the notched oysters was significantly greater, 2.27 times, than in control oysters (*P* = 0.021) while it was 1.72 times that of the abraded oysters (*P* = 0.248). The Spearman’s rank order correlation analysis showed a significant positive correlation between the expression of dominin and that of segon with a correlation coefficient *r*_s_ = 0.752, *P* < 0.001.

### Differences in Dominin and Segon Gene Expression Between Younger and Older Oysters

**FIGURE 10:**
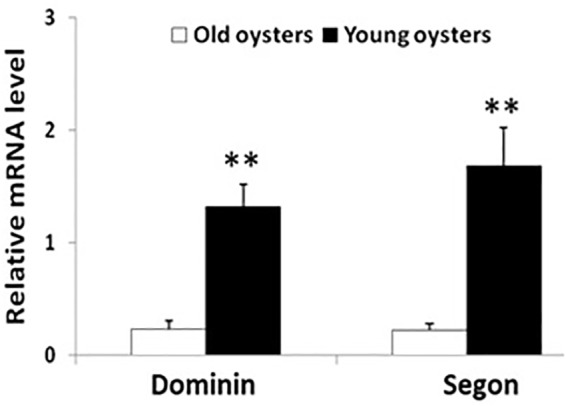
Relative mean ± standard errors expression of dominin or segon gene in hemocytes of 30 months compared to 6 months old eastern oysters (*n* = 5). Asterisks indicate a significant difference (*P* < 0.01).

The relative levels of mRNA of both dominin and segon in hemocytes in 6-month-old oysters were significantly greater (*P* < 0.01) than in 30-month-old oysters (Figure 10).

## Discussion

The native state of dominin and segon in the plasma and their potential involvement in shell formation in eastern oysters were studied. Isolation by density gradient centrifugation and observation by transmission electron microscope revealed that the oyster plasma and extrapallial fluids contained numerous particles relatively homogenous in morphology. Polyacrylamide gel electrophoresis revealed that the particles contained three major proteins matching those observed in plasma and those extracted from shell extracts. Moreover, the expression of dominin and segon genes in hemocytes increased upon initiation of shell repair and were significantly greater in younger oysters. These results indicated that dominin, segon and perhaps some yet to be identified plasma proteins likely associate to form a multiprotein particle in hemolymph and extrapallial fluids and play a role in the initiation of oyster shell formation.

Only a few dozen protein spots were observed after 2-DE of eastern oyster plasma with a few major protein spots being dominant. This result conformed with SDS-PAGE findings that total plasma protein was composed of approximately 50% dominin and 20% segon (Itoh et al., 2011; Xue et al., 2012). The 2-DE profile of eastern oyster plasma was also similar to that of several other bivalves in the number of visualized protein spots (Kania et al., 1980; Suhr-Jessen and Rasmussen, 1988; Oliveri et al., 2014). The low number of protein spots detected may partly be explained by the limited sensitivity of Coomassie blue staining used in our study. Silver staining would have likely revealed additional protein spots as observed for plasma of other bivalves (Simonian et al., 2009a,b; Fernández-Boo et al., 2016; Franco-Martínez et al., 2018). However, the key factor preventing the detection of many more protein species in bivalve plasma is likely related to the limitation of the dynamic range of 2-DE. Unfortunately, even more versatile techniques such as the Orbitrap mass spectrometry can only generate accurate proteomic data for proteins with a dynamic range of about 10^3^ (Yates et al., 2009). On the other hand, most oyster plasma proteins are present at very low concentrations. Xue et al. (2004, 2006) for example, purified only 1 mg of cv-lysozyme 1 from 4.2 g plasma proteins and less than 1 mg of the protease inhibitor cvSI-1 from nearly 40 g total plasma protein. This difference in concentration between dominin and cvSI-1 already represents a dynamic range of over four orders of magnitude and it is likely that many proteins are present in plasma at a lower concentration than cvSI-1. Proteomic analyses of bivalve plasma in future studies will therefore require pre-fractionation to exclude major proteins to gain better insights into the lesser abundant plasma proteins as was done for analyzing plasma or serum proteins of other animals (Tam et al., 2004; Linke et al., 2007; Zolotarjova et al., 2007; Jmeian and Rassi, 2009; Pernemalm et al., 2009). Nevertheless, our findings suggest that the dominance of plasma proteome by a few major enigmatic proteins is a common phenomenon in bivalves.

Following size exclusion chromatography, a single fraction eluted as the first absorbance peak contained most plasma protein and indicated a homogenous large size. At the same time, most plasma protein could be sedimented by ultracentrifugation and the precipitated plasma protein was recovered in a narrow CsCl density zone after isopycnic centrifugation and observed as relatively homogenous particles of about 25 nm in diameter. These protein particles are comparable in morphology to pernin particles purified from the plasma of the New Zealand green-lipped mussel but larger than cavortin particles purified from the Pacific oyster plasma, which was reported to be 15 nm in diameter (Scotti et al., 2001, 2007). Protein particles have also been identified in several other bivalve species. A calcium-binding phosphoprotein particle of 30–40 nm in diameter was sedimented by centrifugation at 100,000 *g* for 60 min from the physiological fluid” of several clam species that belong to the subclass Heterodonta (Marsh and Sass, 1983, 1984, 1985). This particle was not found using the same technique in bivalves belonging to the subclass Pteriomorphia, which includes ark clams, oysters, scallops and mussels (Marsh and Sass, 1985). However, protein particles, although smaller in size as compared with the Heterodonta phosphoprotein particles, were later isolated from the plasma of three Pteriomorphia bivalves, the New Zealand green-lipped mussel, the blue mussel and the Pacific oyster by centrifugation at 250,000 *g* for 2.5–3 h (Scotti et al., 2001, 2007). We found that under the centrifugal conditions for the isolation of phosphoprotein particles (i.e., 100,000 *g* for 60 min) less than 10% of oyster plasma protein was precipitated. This may explain the initial failure to isolate protein particles in the Pteriomorphia species (Marsh and Sass, 1985). In addition to the presence in circulating plasma, protein particles are found in the extrapallial fluid of bivalves (Marsh and Sass, 1983; Hattan et al., 2001; Yin et al., 2005). We also purified the morphologically identical protein particles in eastern oyster extrapallial fluid (data not shown). Therefore, it may be a common phenomenon among bivalves that major proteins form multiprotein particles representing a functional unit in the hemolymph.

SDS-PAGE analyses of the protein particles purified from eastern oyster plasma and extrapallial fluid uncovered three major proteins corresponding to dominin, segon, and an unidentified protein of about 70 kDa as estimated on the basis of electrophoretic migration behavior. In addition, native-PAGE isolated two aggregates from the purified protein particles and both were characterized in SDS-PAGE to be composed of the same three major proteins identified directly from the particles. It is thus likely that dominin, segon and the 70 kDa-unknown protein assemble to form a multiprotein complex, which in turn self-assemble to form protein particles. This belief is consistent with the previous speculation that segon is present in oyster plasma by complexing with other plasma proteins (Xue et al., 2012). Our findings that eastern oyster plasma protein particles are composed of heterogenous proteins appears to differ from results of some previous studies. The phosphoprotein particles of heterodont bivalves were reported to be dissociated into non-identical subunits after removal of the mineral component with EDTA but all the subunits were polymers of a single phosphoprotein molecule (Marsh, 1986b, 1990). Likewise, plasma particles isolated from New Zealand green-lipped mussels and Pacific oysters were reported as polymers of pernin and cavortin respectively (Scotti et al., 2001, 2007). However, when the photographs of SDS-PAGE of the proteins of cavortin or pernin particles presented by Scotti et al. (2001, 2007) are examined carefully, at least two protein bands can be seen. For example, cavortin purified by Scotti et al. (2007) contains cavortin and a protein that is at a similar position to the 66 kDa BSA band used as standard and to the protein later identified as Cg-segon by Xue et al. (2012) based on amino acid sequence similarity with Cv-segon. In European flat oysters, Xue et al. (2012) have also identified the second most abundant plasma protein and named this protein Oe-segon based on N-terminal sequence similarity with Cv-segon. Furthermore, both Cg-segon (∼66 kDa) and Oe-segon (∼44 kDa) had higher SDS-PAGE derived molecular weights than Cv-segon (∼39 kDa). Intriguingly, an earlier study by Morga et al. (2011) reported the cDNA of a protein named “Oe-EcSOD” which upon our further examination encompasses the full Cv-segon sequence and a small part of the dominin sequence. Alignment of the amino acid sequences of Oe-EcSOD, Cv-segon, and dominin using the Clustal omega program indicated that the first 100 amino acids from the N-terminal of Oe-EcSOD are similar to those of Cv-segon with an identity of 48.54% and the following 164 amino acids match those of dominin with an identity of 60.62% (Analysis not shown). This finding suggests that the segon gene may have evolved in *O. edulis* and likely other oyster species by recombination with a dominin homologous gene, reflecting the functional significance of the cooperation between the two proteins. It can also further support the hypothesis of the formation of a multiprotein complex in eastern oysters which have separate genes for expressing segon and dominin. In any case, proteins associated with the plasma and extrapallial fluid particles to date share two remarkable characteristics: secretion by hemocytes and capacity to bind calcium and other metals (Marsh and Sass, 1984; Marsh, 1986a, 1990; Scotti et al., 2001, 2007; Renwrantz and Werner, 2008; Itoh et al., 2011; Xue et al., 2012). These common features may entail functional similarities.

Our results showed that dominin, segon and a third protein identified in eastern oyster plasma and plasma protein particles were extracted from decalcified new shell growth, clearly demonstrating their integration in the shell organic matrix. In addition, the increase of dominin and segon gene expression in hemocytes concomitant with the start of new shell growth after shell damage further indicate their role in the initiation of shell repair. Furthermore, the significantly higher levels of dominin and segon gene expression in faster growing younger oysters than in older oysters also suggest the involvement of the two proteins in shell growth. Interestingly, dominin and segon had the same pattern of gene expression in response to shell damage and the expressions showed a significant positive correlation. These results indicate that the two proteins function in tandem and thus support the speculation that they aggregate as a multiprotein complex in the form of particles. At the same time, the positively correlated expression of dominin and segon implies a coordinated control of gene expression machinery that should be further studied. In heterodont bivalves, phosphoprotein particles have been proposed to serve as calcium transporters and reservoirs in shell development (Marsh and Sass, 1984). Likewise, the major calcium binding protein (EP) in extrapallial fluid of blue mussel, which is the same protein as HRG in plasma, is believed to be involved in forming the shell matrix (Hattan et al., 2001; Yin et al., 2005). The major plasma proteins in eastern oysters may therefore also function in calcium transport or extracellular organic matrix assembly or in both. It remains to be determined how the major plasma proteins are integrated into the shell matrix. One possibility could be that the proteins or protein particles in the extrapallial fluid self-assemble to form the organic matrix as suggested by the drastic changes in secondary structures of the major calcium binding protein in blue mussel (Yin et al., 2005). Earlier studies have also speculated that Zn and Cu, which accumulate at high concentrations in hemocytes of eastern oysters, are released from these cells and interact with plasma proteins initiating extracellular “clot” formation (Fisher, 2004). Alternatively, the proteins could be deposited by hemocytes as hemocytes are observed to play a role in calcium carbonate crystal formation and shell mineralization (Mount et al., 2004; Cho and Jeong, 2011; Johnstone et al., 2015; Li et al., 2016). Further studies on the dynamic changes in calcium and other metals binding to proteins in hemocytes, plasma, extrapallial fluid and the shell extracellular organic matrix during shell growth are needed before a conclusion can be drawn especially considering the recent study indicating that calcium is transported mainly in the ionic form in the hemolymph of Pacific oysters (Sillanpää et al., 2016).

In summary, dominin, segon and perhaps some other plasma proteins appear to form a protein particle homogenous in morphology and heterogenous in protein composition. These proteins may first bind to form a multiprotein complex and then associate to form the protein particle. They likely participate in the initiation of oyster shell formation either in the form of separated proteins, protein particles or protein particle aggregates although the specific and sequence of processes that are involved remain to be elucidated. It is also important to note that comparable major plasma proteins such as HRG (i.e., also named HIP, EP, SBP1) in blue mussel have been extensively studied for their role in the transport of heavy metals (Renwrantz et al., 1998; Nair and Robinson, 1999, 2001a,b; Devoid et al., 2007; Robinson, 2013). Additionally, recent studies have confirmed the role of different hemocyte types in the accumulation of cooper, zinc and calcium in the Hong Kong oyster, *Crassostrea hongkonggensis* (Weng et al., 2017; Wang et al., 2018). The compartmentalization of metals and major plasma proteins that are produced by hemocytes and their interactions in relation to shell formation therefore need to be further investigated. In addition, the potential role these major plasma proteins may play in oyster metal detoxification and metal accumulation should also be addressed in future research.

## Author Contributions

QX designed the research, conducted the protein related experiments and data processing, and drafted and revised the manuscript. J-PB conducted the oyster shell damage experiments and measured and analyzed all gene expressions. JL designed part of the research, mentored J-PB work, and critically revised the manuscript.

## Conflict of Interest Statement

The authors declare that the research was conducted in the absence of any commercial or financial relationships that could be construed as a potential conflict of interest.
